# Effectiveness of an Online CBT-I Intervention and a Face-to-Face Treatment for Shift Work Sleep Disorder: A Comparison of Sleep Diary Data

**DOI:** 10.3390/ijerph16173081

**Published:** 2019-08-24

**Authors:** Lukas Peter, Richard Reindl, Sigrid Zauter, Thomas Hillemacher, Kneginja Richter

**Affiliations:** 1 University Clinic for Psychiatry and Psychotherapy, Paracelsus Medical University, 90419 Nuremberg, Germany; 2 Faculty for Social Sciences, University of Applied Sciences Georg Simon Ohm, 90402 Nuremberg, Germany; 3 Faculty for Medical Sciences, University Goce Delcev, 2000 Stip, Republic of North Macedonia

**Keywords:** chronobiology, shiftwork, insomnia, cognitive behavioral therapy, telepsychiatry, occupational health

## Abstract

In western societies, about one in six employees works in shifts. Shiftwork is associated with a number of poor somatic and psychological health outcomes, especially sleep issues. Higher rates of absenteeism and accidents in the workplace are possible consequences. Still, prevention programs and treatment options that are specifically tailored to shift-workers’ needs are rare. We devised a 4-week online cognitive behavioral therapy for insomnia (CBT-I) intervention (*n* = 21) and compared sleep outcomes to a face-to-face outpatient treatment for shift-workers (*n* = 12) using a sleep diary and the Epworth Sleepiness Scale (ESS). In the online sample, measures also included the World Health Organization wellbeing questionnaire (WHO-5) and the Insomnia Severity Index (ISI). In the outpatient sample, the Beck Depression Inventory (BDI-II), the Montgomery–Åsberg Depression Rating Scale (MADRS), and the Pittsburgh Sleep Quality Index (PSQI) were administered. Results showed significant improvements in sleep efficiency by 7.2% in the online sample and 7.7% in the outpatient sample. However, no significant difference was found in the rate of improvement in sleep efficiency across four weeks of treatment between the samples. In the online sample, the wellbeing (WHO-5) and insomnia symptoms (ISI) scores were significantly improved following the CBT-I intervention (*p* < 0.004 and *p* < 0.002 respectively). In the outpatient sample, symptoms of depression (BDI-II and MADRS scores) and insomnia symptoms (PSQI scores) improved significantly following the CBT-I intervention. In summary, CBT-I significantly improved sleep efficiency in both the online and outpatient samples, in addition to wellbeing, symptoms of insomnia, and depression. The findings of this study demonstrate online CBT-I as a feasible approach for treating insomnia in shift-workers. Future randomized controlled trials are needed.

## 1. Introduction

Across western societies, about one sixth of the working population is on a shift schedule [1,2,3]. Shiftwork includes any professional activity where working hours change according to a defined cycle, for example daily or weekly [4]. Working shifts is associated with increased risks of cardiovascular diseases, gastrointestinal disorders, breast cancer, as well as sleep disorders [5,6,7,8].

Multiple psychosocial, physiological, and behavioral mechanisms may contribute to causal connections between shiftwork and different disorders. On a psychosocial level, shiftwork is associated with reduced flexibility, less control over working conditions, impaired work-life-balance, and recovery from work [6]. These factors can be interpreted as reduced job control [9] and as an effort–reward imbalance [10]; both models have been used in the past to causally connect poor working conditions with cardiovascular risk factors such as hypertension or atherosclerosis [6,11]. Behaviorally, a number of potentially unhealthy habits are associated with shiftwork. This includes smoking, dietary cholesterol consumption, weight gain, and binge drinking [6,12]. The most prominent behavioral factor linking shiftwork and disease, however, is sleep. Shiftwork disrupts circadian rhythms and melatonin secretion, which are in turn associated with increased risk of breast cancer [5,13]. Other consequences of having to work against internal rhythms include sleep deprivation, insomnia, and daytime sleepiness [5,13,14,15]. One in four shift-workers and one in three night-workers suffer from clinically relevant symptoms of sleep disorders [16,17]. Difficulties initiating or maintaining sleep and daytime sleepiness that persist for more than three months and are clearly linked to the individual shift rotation may be diagnosed as “shift work sleep disorder” according to the International Classification of Sleep Disorders [18]. The associated impairments in daytime functioning result in increased risks of accidents in the workplace and on the commute, which add up to billions of dollars in damage and healthcare costs every year in the US alone [19,20].

Seeing as shift work is indispensable in a number of different industries, programs for prevention and intervention that are especially tailored to the needs of shift-workers are needed [21]. This includes ergonomic redesign of shift schedules and work environments. Fast forward shift rotation (2 morning-, 2 evening-, 2 night-shifts) with adequate time off between shifts (>11 h) and multiple days off after night shifts are recommended [21]. There is some evidence that the application of bright artificial light between 7000 and 12,000 Lux during night-shifts and wearing sunglasses on the morning commute home might improve circadian adaptation and wakefulness at work [15,22,23]. Allowing short naps during night-shifts could potentially also improve wakefulness and performance [21]. There is however some conflicting evidence for most of these preventive measures [21], so providing clear recommendations presents a challenge for researchers.

Data on possible treatments for shift work-related sleep disorders is scarce and as of today limited to the administration of melatonin [23,24,25,26], other sleep-inducing medications [27], and relatively short interventions relying on elements from cognitive behavioral therapy for insomnia (CBT-I) [28,29].

CBT-I treatments typically include sleep restriction, stimulus control, psychoeducation, relaxation techniques and cognitive restructuring [30,31]. Sleep restriction refers to the creation of voluntary mild sleep deprivation by limiting time in bed to the average actual sleep time, thus increasing homeostatic sleep pressure and decreasing sleep fragmentation. If a patient has typically spent nine hours in bed but has only slept for six of those before beginning treatment, their time in bed will be limited to six hours per night, until their sleep efficiency has increased to a satisfying degree, typically 85% or higher. Time in bed is then gradually increased until sleep efficiency stagnates at around 85%. Stimulus control refers to establishing a regular sleep-wake schedule and using the bed only for sleep and sexual activity, thus retraining the patient to disassociate the sleeping environment with activities such as smartphone use, television, or work. Relaxation techniques, such as progressive muscle relaxation (PMR) or breathing exercises, are used to reduce physiological hyperarousal and intrusive thoughts. Psychoeducation and cognitive restructuring entail providing guidelines regarding helpful versus detrimental thoughts and behaviors as well as modification of faulty beliefs about sleep, insomnia and its consequences. Treating shift-workers with CBT-I requires modification to some of those elements, especially stimulus control and sleep restriction [28], as sleep-wake rhythms can never be completely regular in this population.

CBT-I has been shown to be at least as effective as sleep medications in increasing sleep efficiency and total sleep time in the short-term and significantly more effective in the long-term [32]. CBT-I is thus considered a standard treatment for chronic insomnia [33]. There are commercial providers specializing in workshops and training programs for shift-workers or in designing and implementing shift schedules [34]. However, to our knowledge, the evaluations of these programs are rarely published.

The outpatient department of the University Clinic for Psychiatry and Psychotherapy in Nuremberg, Germany offers a treatment program for shift-workers with sleep disorders where six sessions of CBT-I are combined with bright light therapy and actometric diagnostics.

Personnel expenditures and waiting times are high for single-outpatient treatments like this. Randomized controlled trials showed that a transfer of CBT-I from outpatient to online settings is principally possible and effective [35,36,37,38,39,40], especially when the delivered contents are complemented by individual therapist support [41,42,43,44]. One study also showed promising results for a 4-week self-help CBT-I intervention augmented by audio files in shift-workers [29]. Thus, we designed, implemented and evaluated a short online intervention to deliver basic CBT-I elements via four e-mail contacts.

This study aimed to compare the sleep outcomes of CBT-I between the online and outpatient samples. Secondarily, in the online sample, the study assessed if CBT-I improved wellbeing as measured by the World Health Organization wellbeing questionnaire (WHO-5) [45], symptoms of insomnia as measured by the Insomnia Severity Index [46], and daytime sleepiness as measured by the Epworth Sleepiness Scale (ESS) [47]. In the outpatient sample, the study assessed if CBT-I improved symptoms of depression as measured by the Beck Depression Inventory (BDI-II) [48] and the Montgomery–Åsberg Depression Rating Scale (MADRS) [49], insomnia symptoms as measured by the Pittsburgh Sleep Quality Index (PSQI) [50], and daytime sleepiness as measured by the ESS [47].

## 2. Materials and Methods

Potential participants for the online intervention were informed and screened by their company physician in six locations of the partnering Robert Bosch GmbH across Germany, resulting in 50 recruited participants, of which 21 went on to complete the entire online treatment course. The information material contained a self-report screening questionnaire asking about nine characteristic symptoms of sleep disorders: problems initiating and maintaining sleep, long periods of wake after sleep onset, feeling tired after getting up and during the day, feelings of inner tension, sleep-related avoidance behaviors and rumination, as well as suffering from sleep problems for more than three months [51]. Participants that confirmed at least six of those symptoms received an individual, anonymized access code to the online treatment platform from their company physician. Alternative treatment options were explored with all other participants. In case of sustained interest, participating in the online treatment was still possible.

Upon registration on the online platform, using the access code, participants received a welcome message containing sociodemographic questions and were administered three questionnaires for evaluating the effectiveness of the CBT-I intervention (WHO-5, ISI, ESS), in addition to a sleep diary (see below). Participants who had exceeded the clinically critical scores (WHO-5 < 13; ISI > 14; ESS > 10) were encouraged to seek outpatient treatment and be diagnosed by a qualified physician. On a transitional basis, online treatment remained possible in these cases. Psychometric measurements were repeated upon completion of the treatment process. Sociodemographic questions included age, gender, composition of household, employment status, and shift schedule.

Online participants were administered a sleep diary (see Table 1), developed based on experiences from outpatient treatment of shift-workers and recommendations of the German Association for Sleep Medicine [52]. Participants were encouraged to complete it daily following waking. The diary contained five evening questions, such as daily amount of alcohol consumed and amount of daytime sleep, as well as ten morning questions, such as bedtime, wake after sleep onset, and total sleep time. Diaries were filled out for one week at a time and transmitted at least four times over the course of the treatment. Participants were able to attach a message with supplementary information about the shifts they had worked, days off, etc.

The usual six sessions of outpatient CBT-I [31] were condensed to four e-mail contacts for the purpose of a more economic approach (see Table 2). Every contact was used to transmit individual feedback about last week’s sleep diary and consequences for the individual sleep restriction program. During the entire program, participants were anonymous to the CBT-I professional with no possibility of connecting the individual access code to any personal information. However, participants were able to interact with the professional and the researchers via phone or e-mail, if they had technical difficulties or specific questions about the CBT-I modules.

In order to recruit potential participants for the outpatient sample, the physicians in our outpatient clinic for sleep disorders screened all new patients referred to us for shiftwork-related sleep disorders between 2015 and 2018. If the diagnosis of shift work sleep disorder was made, patients were informed and written consent was obtained. Patients then received six face-to-face, one on one sessions of CBT-I with a trained psychologist as well as actometric diagnostics between the first and second session and bright light treatment between the third and fourth session (see Table 2). Sessions took place in our clinic every 14 days, except for the last session, which was scheduled 28 days after the fifth. Participants in the outpatient sample also completed sleep diaries between sessions. For the outpatient sample, interaction with their CBT-I professional or the researchers via phone or email was also possible between sessions. At the beginning of the first and the end of the last session, outpatients completed the ESS, BDI-II, MADRS, and PSQI questionnaires.

Total sleep time, total bed time and sleep efficiency were computed as weekly averages from the sleep diaries. The ESS consists of 8 items, which are self-rated and scored from 0–3 [47]. Total scores thus range from 0 to 24, with higher scores indicating higher daytime sleepiness. The WHO-5 is a self-report scale consisting of 5 items assessing positive well-being on a scale of 0 to 5 [45]. The total score thus ranges from 0 to 25, which is multiplied by 4 to yield percentile scores between 0 and 100. The ISI is a 7-item self-report instrument assessing insomnia severity [46]. Each item is rated on a 5-point scale, resulting in possible scores ranging from 0 to 28 with higher scores indicating more severe symptoms. The BDI-II is a 21-item self-report screening measure for depressive symptoms [48]. Items are rated on scales from 0 to 3 with higher scores indicating more severe depression. The total score can range from 0 to 63. The MADRS is a 10-item clinician rating for symptoms of depression [49]. Each item can be scored on a scale of 0 to 6, yielding total scores between 0 and 60 with higher scores representing higher levels of depression. The PSQI is a 19-item self-report questionnaire for subjective sleep quality [50]. The items are combined into 7 sub-scores with possible values ranging from 0 to 3. These scores are added for a total score between 0 and 21 with higher scores representing lower sleep quality. 

Comparison of sleep outcomes from sleep diary data between the online and outpatient samples as well as comparisons between pre- and post-intervention data were computed using *t*-tests and repeated measures analysis of variance in the IBM SPSS Statistics software [53].

## 3. Results

The first contact of the online treatment program was initiated with 50 participants, aged between 20 and 63 years (M = 43.4; SD = 10.5). The sample was predominately male (74%), living with a spouse and/or children (78%), and working full time (94%). Only 36% of online participants reported working shifts during the entire treatment period. The rest of the sample either was on vacations or had changes in scheduling during the program or failed to report their shift schedule. Complete data including at least four sleep diaries as well as pre- and post-measurements of the ESS, WHO-5, and ISI were obtained for 21 online participants. Between being first contacted by a new client and sending off the last message in the online CBT-I intervention, an average of 98.42 days passed (SD = 43.63).

At baseline, 45% of the online sample reported critical sleep efficiency (<85%), calculated weekly as the quotient of total sleep time divided by total bedtime (see Table 3). 46% of the online sample reported critical scores regarding daytime sleepiness (ESS > 10) and 74% reported critically impaired well-being (WHO-5 < 13). At least subclinically relevant scores regarding symptoms of insomnia (ISI > 7) were reported by 96% of the sample.

**Figure 1:**
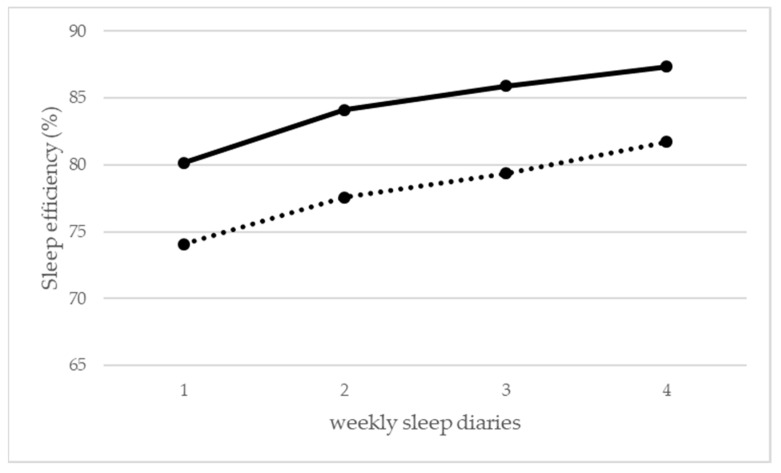
Average sleep efficiency (%) across the first four sleep diaries. Online sample (solid line) and outpatient sample (dotted line).

Following the online CBT-I intervention, analyses of variance showed linear improvements in sleep efficiency over the four sleep diaries (*p* < 0.01; Figure 1) with an average improvement of 7.2% from the first to the last week. The ESS score and total sleep time did not change significantly between the first and the fourth week (Table 3). Symptoms of daytime sleepiness did not change significantly. ISI (*p* = 0.002) and WHO-5 scores (*p* = 0.004) improved over the course of the program (see Table 3). Between the second and the fourth weekly sleep diary, total sleep time per night increased linearly by an average of 25.5 min (*p* = 0.007; one-tailed), independent of shiftwork status. The first week of CBT-I treatment was omitted from this analysis, as it contained sleep restriction, which is usually accompanied by an initial drop in total bedtime. However, sleep times did not change significantly between the first and second sleep diary.

For the outpatient sample, 20 patients were recruited and introduced to treatment. Participants were aged between 26 and 58 years (M = 48.1; SD = 9.4). Like the online sample, the outpatient sample was mostly male (80%), living with a spouse and/or children (80%), and working full time (90%). The outpatient sample also reduced in size due to a high dropout rate. Reasons for dropout included prolonged sick leave, changes in medication, and unexcused absence. At least the first four sessions of CBT-I and corresponding sleep diaries were completed by 12 participants in the outpatient sample.

In the outpatient sample, 67% of participants reported critical sleep efficiency at baseline. 42% reported critical daytime sleepiness on the ESS. At least mild symptoms of depression were reported by 68% of the outpatient sample on the BDI-II and by 100% on the MADRS. At least subclinically relevant symptoms of insomnia on the PSQI were reported by 100% of the outpatient sample.

Analyses of variance also showed linear improvements of sleep efficiency in the outpatient sample (*p* < 0.001) by an average of 7.7% between the first and the fourth week of treatment. The ESS score and total sleep time did not change significantly between the first and the fourth week (Table 3). Symptoms of daytime sleepiness did not change significantly over the course of the outpatient treatment. Following the CBT-I intervention, scores on the BDI-II (*p* = 0.007), MADRS (*p* < 0.001), and PSQI (*p* = 0.007) improved in the outpatient sample (see Table 3).

Comparison of baseline data showed no significant differences between online and outpatient samples in the average sleep efficiency and total sleep time, and ESS scores (see pre-data in Table 3). Although both the online and the outpatient samples showed significant improvement in sleep efficiency following CBT-I, the rates of improvement across four weeks of treatment were not significant differences between them (see Figure 1). The improvements regarding sleep efficiency can thus be largely ascribed to significant reductions in total bed times (see Table 3). 

## 4. Discussion

We piloted an online CBT-I intervention aimed to increase sleep efficiency, reduce symptoms of insomnia, and improve well-being in shift-workers. We then compared their sleep diary data to treatment outcomes in a face-to-face CBT-I outpatient sample of shift-workers. Since online interventions are relatively inexpensive and easily accessible, an effective online CBT-I program could be valuable for shift-workers and their employers. We found that online CBT-I for shift-workers is as effective in improving sleep efficiency as face-to-face treatment.

In online settings, anonymity allowed participants to receive treatment without fear of reprisal (e.g., fear of being taken off their shift schedule by their employer, which would usually be accompanied by a significant cut in their paycheck). In severe cases of shiftwork sleep disorder, establishing regular working hours may be necessary in order to treat the condition effectively. However, most of our shift-working patients do not want to leave their schedule due to financial incentives. Presenting an anonymous treatment option may empower those who previously did not seek help in fear of their employer finding out about their condition.

Improvements in sleep diary measures (i.e., an increase of 7.2% in sleep efficiency in the online sample and 7.7% in the outpatient sample) are in line with the literature where mean increases of about 7% have been reported in online CBT-I interventions compared to waiting list [42]. Whilst the study also reported an increase of 20 min in total sleep time [42], our study did not observe a statistically significant change for this variable. 

As shown by relatively high rates of impaired well-being and symptoms of insomnia in this sample, untreated, at least subclinical sleep disorders affect a relevant part of the working population. As expected from clinical experience and the contemporary literature on CBT-I [55], well-being and symptoms of insomnia improved over the course of the treatment in both the online and the outpatient sample. Daytime sleepiness however did not change significantly in either group. This might be attributed to initial impairments in symptoms of sleepiness during the first weeks of sleep restriction therapy [56,57]. 

Most participants in this study were male, even though women showed a higher prevalence of insomnia symptoms [8] and generally made more use of outpatient mental health services [58]. We assume that, apart from the target population in our partner companies being predominantly male, men might prefer anonymous online settings, such as the one described here, to outpatient therapy options. Data on college students showed no gender differences regarding preference for online versus face-to-face therapy [59] but we suggest that this might be subject to change in other age groups, where women have been found to express more favorable attitudes towards face-to-face treatment than men [60]. Online therapy could thus be used in gender-specific approaches to prevention and treatment of sleep disorders in men.

To our knowledge, the described program is the first evaluation of an online treatment for sleep disorders in shift-workers. One of the limitations of this study is the relatively low number of participants who worked shifts full-time during the entirety of the study. This may be attributed to difficulties in the recruitment process, as well as high dropout rates. The online intervention in this study was not automated but relied on the CBT-I professional to reply to every message manually. This may have led to delays of several days between messages, for example over weekends, which might have created a level of frustration in some participants, who might then have stopped checking their message inboxes on the online platform. Other studies on digital CBT-I interventions have also reported dropout rates of more than 50% [55,61]. Additionally, the shift-work population might be more prone to dropout in programs like this, as shown in other studies [29], as free time is often a scarce resource for shift-workers [6]. Future studies might benefit from close cooperation with partnering corporations, as well as more user-friendly interfaces and short time lags between messages, for example by automating parts of the intervention.

Another limitation in this study concerns its design. The lack of randomization, a control group, or an extended follow-up period severely hinder interpretation and transferability of the results. Randomized controlled trials with long-term follow up measurements are needed in order to be able to make statements about the efficacy of this CBT-I treatment.

Finally, due to different questionnaires used for the outcome measures, we were only able to compare the two samples based on sleep diary data and the ESS. Comparisons of other psychometric measures were not possible. Future studies might thus compare online and face-to-face CBT-I treatments more thoroughly employing a range of self-report measures for symptoms of insomnia and depression.

## 5. Conclusions

According to these preliminary findings, treating shiftwork-related sleep problems in an anonymous online setting appears feasible and effective. Therapist-guided online CBT-I interventions could improve sleep efficiency, well-being, and symptoms of insomnia in shift-workers. Target groups may differ significantly regarding symptom severity between online and face-to-face outpatient settings, suggesting randomization and stratification, for example, by insomnia severity and gender to improve balance of these factors.

## Figures and Tables

**Table 1:** Sleep diary.

Question	Example Answer
**Evening questions**	
Have you had daytime sleep today? If so, please report total sleep time.	Yes, 30 min
Did you take medication for sleep today? If so, what kind?	Yes, Mirtazapine
Have you had alcohol today? If so, what kind and how much?	Yes, 3 glasses of wine
How was your mood before going to bed? (scale of 1 = great to 6 = horrible)	2
How tired were you before going to bed? (scale of 1 = not tired at all to 6 = dead tired)	4
**Morning questions**	
At what time did you go to bed?	23:00
When did you turn of the lights?	23:30
How many minutes did it take you to fall asleep?	20
How often did you wake up last night?	Twice
How many minutes did you lie awake? Don’t count the minutes it took you to fall asleep.	40
What time did you last wake up tonight?	6:00
When did you finally get up?	6:30
How long did you sleep in total?	5:15
How restful was your sleep? (scale of 1 = perfectly restful to 6 = catastrophic)	4
How was your mood after getting up? (scale of 1 = great to 6 = horrible)	5

**Table 2:** Comparison of cognitive behavioral therapy elements in the online and outpatient samples.

Session	Online	Outpatient
**1**	Sleep restriction Instruction via semi-standardized email	Initial examination, diagnosis Semi-standardized clinical interview during face to face session
**Interim**	-	Actometry (14 days)
**2**	Psychoeducation, sleep hygiene Information via semi-standardized email	Sleep restriction Instruction and discussion during face to face session
**3**	Relaxation techniques Information via standardized email, link to online audio resources for progressive muscle relaxation [54]	Psychoeducation, sleep hygiene Information and discussion during face to face session
**Interim**	-	Bright light therapy at home with a loan device
**4**	Concluding remarks, recommendations for the future via semi-standardized email	Relaxation techniques Information and training during face to face session
**5**	-	Cognitive restructuring Information, discussion and training during face to face session
**6**	-	Concluding remarks, recommendations for the future Information and discussion during face to face session

**Table 3:** Psychometric scores and sleep diary data before and after 4 sessions of CBT-I.

	Pre-	Post-	*p*-Value (One-Tailed)
**Online sample (*n* = 21)**			
World Health Organization wellbeing questionnaire (WHO-5)	10.9 ± 3.8	13.9 ± 4.2	0.004
Insomnia Severity Index (ISI)	13.9 ± 3.8	10.6 ± 5.6	0.002
Epworth Sleepiness Scale (ESS)	9.1 ± 3.7	8.2 ± 3.7	0.109
Total sleep time	386.1 ± 58.6	395.1 ± 76.1	0.195
Total bed time	483.7 ± 52.2	458.1 ± 67.1	0.041
Sleep efficiency	80.1% ± 11.6%	87.3% ± 11.1%	0.001
**Outpatient sample (*n* = 12)**			
Beck Depression Inventory (BDI-II)	14.4 ± 10.6	7.3 ± 7.3	0.007
Montgomery–Åsberg Depression Rating Scale (MADRS)	19.2 ± 5.6	8.2 ± 6.5	<0.001
Pittsburgh Sleep Quality Index (PSQI)	11.5 ± 3.9	9.0 ± 4.3	0.007
ESS	9.6 ± 4.6	7.8 ± 3.9	0.124
Total sleep time	349.3 ± 60.9	354.2 ± 64.0	0.377
Total bed time	480.4 ± 61.0	435.8 ± 55.3	0.019
Sleep efficiency	74.1% ± 16.6%	81.7% ± 11.3%	0.026
